# 

**DOI:** 10.1038/sj.bjc.6600375

**Published:** 2002-01-02


A prospective database identified 77 patients who had preoperative radiotherapy for rectal cancer (2500 cGy in 5 consecutive daily fractions in the week preceeding surgery) There were 4 exclusions, 1 neuroendocrine tumour and 3 with incomplete records. For the resulting 73 patients data concerning patient demographics, staging, operation, pathology, morbidity and mortality were recorded by a retrospective case note review.
The median age was 69 years (interquartile (IQ) range 61 – 74) and 18 were female. The median post-operative follow up was 518 days (IQ range 254 – 976). Preoperative radiographic imaging was undertaken in 65/73 patients 44 had a CT scan, 21 a MR scan. In 31 scans there was evidence of perirectal infiltration. Examination under anaesthetic prior to definitive surgery was performed in 32 patients.
In 63/72 operations were performed with curative intent, in 8 the operation was deemed to be palliative in one case the intent was not documented and 1 patient was not operated on. The reasons for palliation were previously undetected liver metastases at laparotomy in 5, in 1 the primary lesion was deemed irresectable, in 1 the primary lesion was locally invasive and in 1 there were irresectable lymph node metastases. Detailed pathology was available in 53 cases. The lateral margin was involved microscopically in 2 patients of whom 1 developed a local recurrence.
Eighteen patients experienced early morbidity, 3 anastomotic leaks, 8 delayed wound healing and 4 urinary retentions, and 1 patient experienced radionecrosis of a perineal wound requiring plastic surgical reconstruction. Local recurrence occurred in 4/72 (5.6%) patients at a median of 354 days post operation (range 74 – 1716); for 2 of these patients surgery had been palliative giving a local recurrence rate of 2/63 (3.2%) for those having potentially curative operations. Distant metatases occurred in 14 patients, 5 at laparotomy, 9 developed metastases at median of 493 days post operation (IQ range 260 – 573). To date there have been 15 deaths at a median survival of 407 days (IQ range 167 – 928), 1 death from locally recurrent disease, 12 from metastatic colorectal cancer and 2 non-cancer deaths (one from postoperative complications).
In conclusion the results of our selective policy indicate a low local recurrence rate for a high risk cohort of patients. The importance of optimal preoperative staging in reducing the number of patients with metastatic disease receiving such treatment is emphasised.

**P67**

**CHEMO-RADIOTHERAPY IN THE MANAGEMENT OF UNRESECTABLE CHOLANGIOCARCINOMA. THE MIDDLESEX HOSPITAL EXPERIENCE 1994-2002;**

S.G. Russell, R. Hughes*, S. J. Rogers, M.Beresford and M.F.Spittle.
Meyerstein Institute of Oncology, Middlesex Hospital W1T 8AA,

Introduction. Cisplatin-based chemo-radiotherapy (chemo-RT) has been advocated as the treatment of choice in the management of a number of malignancies. Its role in the treatment of unresectable cholangiocarcinoma remains undefined. Acute toxicity is a serious concern and may lead to delays in RT which could offset any benefit from the addition of chemotherapy.
Aims. We reviewed the management of patients with inoperable cholangiocarcinoma treated between 1994 and 2002. Of primary interest were overall survival from time of diagnosis, length of inpatient stay, treatment related acute toxicity and delays in treatment.
Methods. The case-notes of forty-one consecutive inoperable cholangiocarcinoma patients were examined. All patients received external beam radiotherapy to the site of disease, either 45 Gy in 25 daily fractions over 5 weeks or 30Gy in 10 daily fractions . Sixteen patients received concurrent chemotherapy. Chemotherapy consisted of 3-weekly cisplatin 100mg day 1 and 3-weekly 5-fluorouracil 750mg days 1 to 3.
Results Of the 41 patients, 28 were male (68%) and 13 female (32%). The average age at diagnosis was 60.4 years (32-77). 33 patients (77%) had histologically proven cholangiocarcinoma. All patients had undergone palliative stenting procedures prior to definitive therapy. The median survival was 14 months (2-31 months) in the chemo-RT group compared with 8 (1-30 months) in the RT alone group. Toxicities in the two groups were similar. Only 1 patient had a grade 3 toxicity of any system, having developed neutropaenic pyrexia following their second cycle of chemotherapy. None of the 16 patients who completed the prescribed course of external beam RT experienced any delays in RT.
Conclusion: Chemo-RT for the treatment of unresectable cholangiocarcinoma is a well tolerated technique without significant additional toxicity and does not result in delays in RT. The overall survival was greater in the combined modality group. Performance status was a selection criterion for chemo-RT. This is in itself a good prognostic factor and may therefore have contributed to the better outcome seen in this group. The cost of chemo-RT and increased time spent in hospital however raise doubts over its routine use. Further randomised data is required.

**P68**

**COLORECTAL CANCER: IDENTIFICATION OF DIFFERENTIALLY EXPRESSED PROTEINS USING A PROTEOMICS APPROACH**

L.C. Lawrie*[1,2], G.I. Murray[2], Departments of [1]Molecular & Cell Biology, [2]Dept. of Pathology, University of Aberdeen, Aberdeen, UK.

Colorectal cancer is one of the most common malignancies in the UK with 30, 000 new cases and 20, 000 deaths from the disease each year. To improve prognosis from colorectal cancer there is an urgent need to identify molecular markers to detect the disease at an early stage where even current treatment regimes would be much more effective. Prognostic markers for colorectal cancer are also vitally required as two tumours may be histologically classified as being at the same stage but these tumours may then go on to have very different outcomes in terms of patient survival. New prognostic markers could predict how a particular tumour is likely to respond to treatment, thus ensuring that patients receive therapy individually tailored to their own requirements. Proteomics is a powerful analytical technique which can help identify diagnostic and prognostic markers for colon cancer by comparing differences in protein expression profiles from normal or tumour tissues. Two-dimensional gel electrophoresis is performed to produce protein expression profiles from fresh frozen paired samples of normal and tumour tissue. Image analysis of these gels identify those proteins which show differential expression, these proteins are likely to be associated with the disease process and can be identified through mass spectrometry and database searching. We have optimised conditions for solubilising the colon tissue and have produced 2D-gel protein expression profiles of patient matched samples of normal and colon tumour tissue, initially selecting tumours from patients with an advanced stage of the disease. The protein expression profiles obtained from the normal and tumour tissue were reproducible not only when replicate samples were taken from the same patient but also when these profiles were compared with samples taken from other patients. Proteins which showed differential expression were excised from the gel and identified through mass spectrometry and database searching. Some of the proteins which showed increased expression in the tumour samples were found to be proteins which are already known to be associated with colorectal cancer progression. We have also identified several novel proteins which are consistently upregulated in the tumour samples. These proteins may be useful as molecular markers for colorectal cancer which could be used to screen patients to detect the disease at an early stage, or as prognostic indicators to determine the likely course of the disease. A possible further application may be to use the proteins which have been differentially expressed as therapeutic drug targets for the development of new chemotherapeutic agents.

**Reference**

Lawrie LC, Fothergill JE, Murray GI., Spot the differences: proteomics in cancer research. *The Lancet Oncology* 2001; 2: 270-77.

**Acknowledgement**
LCL thanks the Scottish Hospital Endowments Research Trust for the award of the Jean V Baxter Research Fellowship.

**P69**

**OUTCOMES OF THE TREATMENT OF HIGH GRADE GLIOMAS - THE CAMBRIDGE EXPERIENCE.**

K.J. Waite*, N.G Burnet and R.V. Bulusu. Dept of Oncology, Box 193, Addenbrooke's Hospital, Cambridge, CB2 2QQ.

**Background.** High grade gliomas have a very poor median survival despite radical treatment. We present the results of 317 adult patients who presented to our unit between October 1996 and August 2001.

© 2002 Cancer Research UK

**Materials and Methods.** Patients were assessed and their treatment was determined by their performance status and age. 158 patients who were fit, with a WHO performance status of 0-1 and aged less than 70 received treatment with a radical intent. Radical radiotherapy was CT planned and a dose of 60Gy in 30 fractions over 6 weeks was prescribed for grade 4 lesions, 54Gy in 30 fractions over 6 weeks for grade 3 lesions, both using 6-8mv photons. 108 patients aged 70 or more and/or with a WHO performance status of 2 or more were treated with palliative intent. Palliative radiotherapy was planned orthogonally using parallel opposed lateral fields and was prescribed to a dose of 30Gy in 6 fractions over 2 weeks. 51 patients with poor performance status received best supportive care alone. Survival was calculated from the date of diagnosis.

**Results.** Those patients who received radical radiotherapy had a median survival of 315 days; those with Grade 3 tumours had a median survival of 1150 days compared to those with grade 4 tumours (glioblastoma multiforme) who had a median survival of 279 days. In 135 patients radical radiotherapy was commenced in a mean of 31.2 days (95% CI 28.1-34.2 days) from being requested. Those patients who received palliative radiotherapy had a median survival of 140 days. In 76 patients palliative radiotherapy was commenced in a mean of 17.6 days from being requested (95%CI 15.2-20 days). Palliative radiotherapy was therefore completed 6 weeks before radical radiotherapy. Those patients who did not receive radiotherapy had a median survival of 43 days.

**Conclusions.** Our results are very similar to other published series and emphasize the need to carefully select patients prior to treatment. The difference in completion time between radical and palliative radiotherapy may be important. Outcome in poor performance patients as expected was very poor. There is a need for alternative strategies to be explored in the treatment of these tumours.

**P70**

**IS IT WORTH REIRRADIATING PRIMARY BRAIN TUMOURS? CAMBRIDGE EXPERIENCE**

Y.L.Rimmer* N.G. Burnet
Department of Clinical Oncology, Addenbrookes Hospital, Cambridge. CB2 2QQ

**Purpose**

The aims were to determine overall survival and progression free survival following reirradiation of malignant glioma, and to assess safety and predictability of outcome.

**Methods**

Eleven patients with a median age of 45.8 years received reirradiation for recurrent gliomas (1997-2001). Eligibility for selection was a performance status of 0-1, a minimum interval of one year from previous treatment and small volume disease. The median interval between the two treatments was 48 months. External beam radiotherapy for the primary treatments was delivered with a variety of methods whilst the reirradiation treatment used a conformal plan with tight margins. The median radiation doses of the first and second treatments were 54Gy and 45 Gy, respectively. The median cumulative biological effective dose (BED) was 181.4 (a/b=2).

**Results**

Subsequent to retreatment, two patients currently remain alive and well with no evidence of recurrence after 41.4 and 23.2 months respectively.The median progression free survival (PFS) of the remaining nine patients was 6.9 months.The six patients who have died had a median overall survival (OS) from completion of retreatment of 19.6 months. Three patients are currently receiving chemotherapy after relapse of disease.Seven patients had surgical intervention at the time of relapse, either biopsy or debulking of tumour. Histological grading remained the same as that on initial presentation in four patients whilst three patients had transformed from grade 3 to grade 4 disease.

Histological grade was not a predictor of relapse. At the time of recurrence the two patients who had grade 2 disease are alive and well but the five patients who had grade 3 disease had a median PFS of 6.9 months and the four patients who had grade 4 disease had a median PFS of 9.2 months. Minimal acute and no late side affects occurred and quality of life remained good even after relapse in the majority of patients.

**Conclusion**

These results support the use of reirradiation in appropriately selected patients with all grades of recurrent glioma. A median PFS of 6.9 months was demonstrated but more interestingly OS determined from the end of the second radiotherapy treatment was 19.6 months.

**P71**

**AN AUDIT OF THE USE OF DOSE VOLUME HISTOGRAMS (DVH's) IN PREDICTING RATES OF RADIATION PNEUMONITIS IN PATIENTS TREATED WITH RADIOTHERAPY TO THE LUNGS**

S J Clenton* M Q Hatton P M Fisher P Kirkbride, All Weston Park Hospital, Sheffield

AIM

Rates of pneumonitis can be correlated to the volume of lung irradiated using 3D treatment planning and DVH's. (1) In Sheffield treatment schedules for thoracic irradiation do not use the convention of 1.8-2 Gy fraction size. However, we have used the experience of Graham et al as a guide to amount of lung irradiated to greater than 20 Gy. This audit aimed to assess if the rates of radiation pneumonitis for our non-conventional fraction sizes are in keeping with those described by Graham et al.

METHOD

This audit has retrospectively reviewed all 3D CT planned thoracic radiotherapy treatments between January 1999 and July 2001. The notes of patients who received doses greater than 36 Gy using an isocentric technique were reviewed for evidence of radiation pneumonitis using the RTOG acute lung morbidity scale. (2)

RESULTS

119 patients were identified and DVH's showed the amount of lung receiving greater than 20 Gy varied from 6% to 51%. 44 were treated with CHART, 48 with radical radiotherapy 54/55Gy in 20 fractions and the remainder received high dose palliative treatment. (Ranging from 36 Gy in 12 fractions to 50 Gy in 20 fractions). Using the RTOG pneumonitis score, 70 score 0, 27 score 1, 4 score 2, 16 score 3 and 2 score 4. Those with evidence of pneumonitis appear to have had a higher percentage of lung treated to greater than 20 Gy.

CONCLUSIONS

The pneumonitis score appeared to be an easy to apply and relevant scoring system which correlated well with descriptions in notes. The rates of pneumonitis were low in the patients treated and appear compatible with the rates described by Graham et al.

REFERENCES

(1) M V Graham et al, 1999, Int. J. Radiation Oncology Biol. Phys., Vol 45, p 323.
(2)R W Byhardt et al, 1993, Int. J. Radiation Oncology Biol. Phys., Vol 27, p537.

**P72**

**SOMNOLENCE SYNDROME FOLLOWING PROPHYLACTIC CRANIAL IRRADIATION IN LIMITED STAGE SMALL CELL LUNG CANCER.**

H.J.H. Taylor*, L.Vini
Royal Marsden Hospital, Sutton, Surrey SM2 5PT, UK.

Introduction: Prophylactic cranial irradiation (PCI) has been shown to offer a small yet significant survival advantage to those patients with limited stage small cell lung cancer (SCLC) achieving a complete response following combination chemotherapy[1]. However, quality of life can be adversely affected by the radiation induced somnolence syndrome, which affects up to 80% of those with primary brain tumours treated with high dose radiotherapy[2].

Aim: To investigate the incidence and duration of somnolence in patients with limited stage SCLC who receive PCI following combination chemotherapy.

Method: Between July 2000 and May 2001, 18 patients (age range 38 – 75 years, mean 57) with limited stage SCLC achieved complete response following chemotherapy and were referred for both consolidation thoracic radiotherapy and PCI. All 18 received consolidation thoracic radiotherapy to the site of disease at presentation (40Gy in 15# over 3 weeks using a 3 field CT planned technique) and PCI (30Gy in 10# over 2 weeks using standard parallel opposed technique). Treatment related toxicity both during radiotherapy and at subsequent follow up appointments was carefully recorded, particularly symptoms compatible with somnolence syndrome.
Results: All patients complained of lethargy during radiotherapy with 8 (44%) requiring regular antiemetics and 3 (16%) starting dexamethasone. 4 weeks following radiotherapy 14 (78%) admitted to symptoms compatible with radiation induced somnolence syndrome, with 8 (44%) taking dexamethasone and 6 (33%) regular antiemetics. The mean duration of lethargy was 3 months (range: 2 weeks to 8 months). In this small series there were no significant predictive factors for prolonged lethargy following treatment.
Conclusion. In this series 78% of patients developed symptoms compatible with the somnolence syndrome following PCI. Further prospective quality of life data is needed. Possible changes in practice include a reduction in radiotherapy dose or the use of prophylactic steroids.

[1] Aupérin A, Arriagada R, Pignon J-P et al. 1999. N Engl J Med; 341:476
[2] Faithfull S and Brada M. 1998. Clin Oncol; 10:250

**P73**

**THE FACILITATION OF ROUTINE CARDIAC SHEILDING IN THE RADIOTHERAPY TREATMENT OF EARLY BREAST CANCER BY INTENSITY MODULATED RADIOTHERAPY (IMRT)**

Le Vay JH*[1], Poynter AJ[1], Mackenzie LM[1].
Suffolk Oncology Centre[1], Ipswich Hospital, Ipswich.UK.

AIM: To investigate whether the acquisition of the facility to use Intensity Modulated Radiotherapy (IMRT) would allow the use of Multi-Leaf Collimation to allow easy and routine cardiac shielding in the treatment of patients with early breast cancer.

METHODS: Cardiac toxicity has been shown to be a cause of long term morbidity and mortality after Adjuvant Radiotherapy in the treatment of patients with early Breast cancer. This has been predominately based on patients treated from an era when precise localisation of treatment fields was more difficult and the Internal mammary chain often treated. (And hence in left sided tumors a significant portion of underlying myocardium was included.) However even with modern CT planned techniques a significant portion of the apex of the heart will be included in the breast tangential fields. The use of a custom made block to shield this portion of the heart is for most centres too time consuming for it to be used routinely in such a commonly treated condition. Modern multi-leaf collimation ( MLC) allows easy and quick individual shielding from the CT plan; however the use of the MLC precludes the use of a wedge due to the position of the MLC leaves. This produces an unacceptable plan. Ipswich hospital is the Varian reference centre for IMRT and the use of IMRT (Electronic compensators) has been introduced in to routine use for the treatment of early Breast Cancer (following an initial pilot study) due to the improvements seen in dose homogeneity. This gave us the option to use the MLC to shield the heart and the IMRT to compensate for the inability to use a wedge. In two patients from the previously reported pilot study in whom the heart was in the desired treatment fields a conventional 3-D plan with MLC shielding was compared to an IMRT plan for the same patient. The plans were compared for adequacy of coverage and dose homogeneity across the target tissue.
RESULTS: The IMRT (Electronic compensator) plan gave an acceptable dose distribution in both patients whereas the non IMRT plan was not considered adequate in terms of dose coverage and homogeneity. The Volume of target tissue receiving greater than 105% of the prescribed dose was reduced by 20% with the IMRT plan. The conventional plan included 10% less of the desired target tissue within the 95-105% isodose range. In both patients the use of IMRT allowed MLC shielding of the heart to be used.
CONCLUSIONS: IMRT (Electronic compensation) allowed the use of MLC shielding of the heart in both cases which was not possible with conventional planning. The use of this technique should be considered in the Routine Radiotherapy of Early Breast cancer in left sided tumors.

**P74**

**LOW DOSE HYPER-RADIOSENSITIVITY - A CLINICAL ASSESSMENT**

Dr Jackie Harney*, Dr Susan Short, Prof. Michelle Saunders
Marie Curie Research Wing, Mount Vernon Hospital, Northwood, Middlesex, UK.

**Background and Purpose**
Recent laboratory studies have demonstrated that some tumour cells are hypersensitive to low doses of radiation (<1Gy/fraction), so called Low Dose Hyper-Radiosensitivity (HRS). There is evidence to suggest that this effect may also exist in normal tissues, including skin
The purpose of this study is to establish whether or not HRS can be demonstrated in the clinic (a) in normal skin and (b) in metastatic tumour nodules, and if it does to determine if there is a therapeutic advantage to using multiple low dose treatments.
**Materials and Methods:**
Following Ethics Committee approval patients with at least two metastatic skin or subcutaneous nodules were recruited to the study. The nodules were measured, then consecutively numbered according to volume and randomised, in matched pairs, to receive either conventionally fractionated radiation treatment (1.5Gy/day), or ultrafractionated radiotherapy (0.5Gy TDS with an interfraction gap of 4hrs). Any unpaired nodule received the ultrafractionated regimen. Both groups were treated for 12 days (total dose of 18Gy).
The tumour nodules were measured in three dimensions on days 0, 4, 8, 12 and 26 and then 2-4weekly until regrowth occurred.
At the first five assessments 3mm punch biopsies of normal skin surrounding one nodule in each treatment arm were taken, fixed and used to assess changes in basal cell density (H&E) and the proliferation markers Cyclin A and Ki67 (immunohistochemistry) with dose.
**Results:**
To date 5 patients have been accrued, giving a total of 29 nodules (12 pairs). Tumour types include leiomyosarcoma, NHL and breast carcinoma.
The results can be divided into two; the effects on the tumour nodules and the effects on the surrounding normal skin.
Effects on nodules :
In one patient (LMS) metastatic tumour nodules exhibited increased growth delay after ultrafactionation (P<0.03). No differences between fractionation regimens have been observed, to date, in the other patients.
Effects on normal skin:
There was no evidence of increased basal cell killing after ultrafractionated treatment in any of the patients. An early reduction in basal cell numbers (days 5-15) was observed, but this was more marked in the conventional arm.
**Conclusions**
Preliminary data presented here suggest that HRS does occur in human tumours at clinically relevant doses. However, the effect was not demonstrable in all patients. These data do not support the existence of HRS in normal skin when 0.5Gy TDS (with a 4hr gap) is compared to 1.5Gy OD, this is contrary to previous data using 0.5Gy once daily.

**P75**

**THE EFFECTS OF LOW-DOSE FRACTIONATED RADIATION ON SARCOMAS- EXPERIMENTAL AND CLINICAL DATA**

S C Short•[1], J Harney[1], F Dopson[2], M I Saunders[1]
[1] Marie Curie Research Wing for Oncology, Mount Vernon Cancer Centre, Northwood UK
[2]The Gray Cancer Institute, Northwood UK

Background: Increased sensitivity to radiation doses below 1 Gy (Hyper-radiosensitivity, HRS) has been confirmed in over 50 human tumour cell lines in vitro. If the same occurs in tumours in vivo it may be possible to exploit this phenomenon clinically by using multiple low-dose treatments in tumours that are resistant to conventional radiotherapy.
Purpose: To examine the response of human sarcoma cell lines to single and multiple low-doses of X-rays in vitro and the response of metastatic sarcoma nodules irradiated in a clinical protocol using a multiple-low-dose per day regime.
Materials and methods: The clonogenic survival of human sarcoma cells (HS633T) was measured in vitro after exposure to single X-ray doses between 0.05 and 5 Gy and after multiple 0.4Gy doses. Accurate measurement of cell survival was achieved by using a Cell Sorter (FACScan, Becton Dickinson, UK) to provide high resolution cell counting. A Pantak unit, producing 240kVp Xrays was used to irradiate sorted cells. The

© 2002 Cancer Research UK

resulting single-dose survival data were fitted to a modified linear quadratic equation, which describes enhanced sensitivity at low doses (Induced Repair equation). In multiple low dose experiments the relative clonogenic survival after 0.4Gy tds and 1.2Gy od daily for 4 days to total doses of 4.8Gy were compared. In the clinical study, following local ethical approval, patients with at least 2 metastatic skin nodules from primary sarcomas were included in a protocol comparing tumour growth delay after 0.5Gy tds or 1.5Gy od for 12 days (total dose of 18Gy). Lesions were measured in 3 dimensions on days 0, 5, 8, 12, 26 then 2-4 weekly. Day 0 measurements were used to pair lesions which were then analysed as matched pairs.
Results: In HS633T cells HRS was demonstrated at doses below 1Gy. In the clinical study 10 skin nodules were analysed in 2 patients with metastatic leiomyosarcoma. In one patient a significant enhancement of tumour growth delay was demonstrated in the low-dose per fraction arm (p=0.003). No significant difference between treatments was demonstrated in the 2nd patient.
Conclusion: HRS can be demonstrated in human sarcoma cells after single and multiple low-dose irradiations in vitro. Preliminary data suggest that the same effect may also occur in metastatic sarcoma deposits but it was not demonstrable in all lesions. This heterogeneity may be due to differences in HRS expression between tumours or an influence of tumour microenvironment.

### P76
### SAFE DELIVERY OF RADIOTHERAPY IN PATIENTS WITH IMPLANTED CARDIAC PACEMAKERS.

S.Santhanam*, S. Bolton, S.Vasanthan, G. Thomas. Dept. of Oncology, University Hospitals of Leicester. Leicester.

**BACKGROUND:** The increasing elderly population and falling cardiovascular mortality is leading to an increase in the number of patients with pacemakers (PM) who need radiotherapy (RT). *In-vitro* studies show that the modern multi-programmable PMs utilising the CMOS circuitry can be adversely affected by therapeutic radiation and by electromagnetic interference from Linear accelerators. (Souliman.1994, Pacing Clin Electrophysiol.17: 270; Venselaar. 1985,Radiother Oncol 3: 81). Despite the potential risk of catastrophic complications in PM dependent patients, the published *in-vivo* data is sparse regarding the safety of RT delivery in patients with PMs. We have set up a database to prospectively collect data on patients with PM undergoing RT.
**PATIENTS, METHODS & RESULTS**: We report 6 cases who were given RT in our department during a 4-month period.(Table). Of these, 5 patients were given palliative RT and 1 was given radical RT (2 had RT to the chest, 2 to Head & Neck (H&N), 1 to axilla and 1 to spleen). The RT fields were arranged to minimize the scattered radiation to the PM. The PM of a 95 yr. old patient was in the radiation field but she was not PM dependent. The patients were monitored with ECGs and pulse oximetry during RT, and PM Clinic checks were arranged following completion of RT. None of the patients experienced any malfunction of the PM during or after delivery of radiotherapy.
**CONCLUSION:** RT can be safely delivered, if direct irradiation of PMs is avoided, adequate monitoring is done during and after irradiation, and the dose to the PM generator is kept below 2 Gy.

| | Sex | Age | Diagnosis | Site of RT & Prescribed RT Dose / Fractions | Calculated Dose to PM |
|---|---|---|---|---|---|
| 1 | M | 79 | Parotid tumour. | H & N. 64 Gy / 32# | 1.3 Gy |
| 2 | F | 85 | Lymphoma. | H &N. 15 Gy / 5# | 0.8 Gy |
| 3 | F | 77 | Myelofibrosis. | Spleen. 4.5 Gy / 9# | <0.5 Gy |
| 4 | F | 95 | Breast cancer. | Axilla. 26.7 Gy / 10# | 26.7 Gy |
| 5 | M | 77 | Lung cancer. | Chest. 15 Gy / 10# | 0.8 Gy |
| 6 | F | 63 | Lung cancer. | Chest. 30 Gy / 10# | 0.6 Gy |

### P77
### EXPERIMENTAL DETERMINATION OF THE OPTIMUM MEANS OF COMBINING TOPOTECAN AND MIBG THERAPY IN THE TREATMENT OF NEUROBLASTOMA

AG McCluskey*, RJ Mairs, M Boyd
Department of Radiation Oncology, University of Glasgow, Cancer Research UK Beatson Labs, Glasgow

Background: Targeted therapy, using radiolabelled meta-iodobenzylguanidine ([$^{131}$I]mIBG) has been shown to cause favorable remissions in neuroblastoma patients when used as a single agent. Long-term cure remains elusive however, and the full potential of this therapy may only be realised when it is combined with other agents. Topotecan (TPT) is a topoisomerase I inhibitor which has been shown to be an effective single agent treatment of refractory neuroblastoma. It has also been shown to act as a radiosensitizer to ionising radiation in vitro. If administered at the appropriate times relative to [$^{131}$I]mIBG delivery topotecan could potentially improve effectiveness by two different mechanisms:- increased concentration of [$^{131}$I]mIBG in neuroblastoma cells and decreased ability to repair targeted radiation damage. Laboratory studies are underway to investigate these effects to determine the most effective means of combining these two treatment modalities.
Methods: The effect of topotecan pretreatment on [$^{131}$I]mIBG uptake was studied in vitro using cultured neuroblastoma cells. Parallel studies on NAT expression were studied using real-time RT-PCR on RNA isolated from pre-treated cells and tumours of mice treated with TPT. Also, the ability of TPT to potentiate radiation-induced toxicity of [$^{131}$I]mIBG in neuroblastoma cells, was examined by clonogenic assay performed on SKNBE(2c) cells exposed to topotecan and [$^{131}$I]mIBG simultaneously. The effects of combination therapy on the DNA of neuroblastoma cells were examined by TUNEL and SCGE. Finally, topoisomerase I levels were evaluated in the neuroblastoma cell lines SKNBE(2c), SKNSH, SKNMC and SHSY5Y.
Results: Cells pre-treated with TPT have shown a 3 fold increase in uptake of [$^{131}$I]mIBG compared to untreated cells. Real-time RT-PCR indicates a 2-fold increase in NAT gene expression. Isobologram analyses of preliminary radiosensitization experiments suggest that combined TPT and [$^{131}$I]mIBG therapy produces synergistic killing in neuroblastoma cells. TUNEL and SCGE analyses of DNA strand breaks show an increase in double strand breaks in cells dosed with TPT and [$^{131}$I]mIBG in combination, relative to either untreated cells, or cells treated with either agent alone**.** Relative topoisomerase I levels in neuroblastoma cell lines were identified as follows: SKNBE(2c) - 2U/ul; SKNMC - 3.37U/ul; SKNSH - 3.05U/ul; SHSY5Y - 1.56U/ul.
Conclusions: These results are encouraging for the use of topotecan in combination with [$^{131}$I]mIBG in the treatment of neuroblastoma.

### P78
### TOTAL SKIN ELECTRON TREATMENT FOR MYCOSIS FUNGOIDES – CLINICAL OUTCOME OF A MODIFIED VERSION OF THE MANCHESTER ARCING BEAM TECHNIQUE.

D. Wilson*, N. Thorpe, P. Chung, A. Stevens, J.A. Mills, R. Grieve, S Colligan.
Walsgrave NHS Trust, Clifford Bridge Road, Coventry CV2 2DX. UK.

**Introduction:** Total Skin Electron (TSE) treatment is an effective treatment for Mycosis Fungoides (MF), with reported response rates of 70-90%. Several TSE techniques have been described, most requiring extensive linear accelerator or room modification. A modified version of the Manchester arcing beam TSE technique, using 6 MeV electrons from a standard Philips SL25 linear accelerator has been developed, in an attempt to improve the dose distribution observed with the Manchester Technique, where lateral dose fall of is a problem. Patients were inclined at 30$^{o}$ to the horizontal during treatment, and the standard electron scattering foil was modified to further increase the angle of incidence of the beam. The prescribed dose was 24 Gray in 12 fractions, treating 2 quadrants daily for 4 days per week, overall treament time being 3 weeks[1].
**Method:** Between March 1996 and November 2000, 20 patients with extensively pre-treated stage Ib – IV MF (30% with stage IV disease) were treated using a modified version of the Manchester arcing beam TSE technique. Clinical data pertaining to response, time to progression and toxicity were obtained retrospectively from case notes.

**Results:** Mean time from diagnosis to treatment was 7 years (range 9 months – 15 years). Follow-up was for a minimum of 8 months, with a mean of 17 months. Response data were available for 17 patients (85%). The overall response rate was 87.5% (25% complete response and 62.5% partial response). To date, 11 patients from the study cohort have died. Mean survival subsequent to TSE was 15.3 months. The mean time to progression was 4 months (range 7 weeks to 15 months – with no sign of relapse to date in one patient after 15 months of follow-up). Residual disease after TSE was successfully treated with local radiotherapy and/or PUVA in all cases. Common sites of disease recurrence were the axillae (4 patients), upper arms (3), lower arms (3) and feet (3). Thus, common sites of relapse were areas known to receive a lower dose in dosimetry studies[1.]
**Toxicity:** Two deaths occurred shortly after treatment (MRSA skin infection and septicaemia in 1 patient and hypostatic pneumonia in a second). All patients completed treatment once started, although in 2 patients it was necessary to interrupt treatment temporarily due to severe skin reaction. Data for skin reactions were available for a subset of 12 patients –67% suffered WHO grade 3/4 toxicity and 17% Grade 2.
**Conclusion:** The modified arcing beam technique demonstrated a high palliative response rate (87.5%) in patients with extensively pre-treated disease. A short mean time to progression (4mo) and severe skin toxicity were observed. A Betatron electron source is under commission and is expected to give improved dosimetry and clinical outcome.
References: 1. Chung P, Thorp NC, Wilson DC. Total Skin Electron Treatment for Mycosis Fungoides – The Coventry Experience. *2001.Br J Cancer; 85(Suppl 1):70.*

**P79**

**PROLIFERATIVE VILLONODULAR SYNOVITIS TREATED SUCCESSFULLY WITH CONSERVATIVE SURGERY AND EXTERNAL BEAM RADIOTHERAPY**

S. Mawdsley*[1], R. Glynne-Jones[2], S. Cannon[3], T. Briggs[3] and S. Short[2]. [1]The Gray Cancer Institute & [2]Marie Curie Research Wing, Mount Vernon Hospital, Northwood, Middlesex HA6 2RN, UK. [3]The Royal National Orthopaedic Hospital, Brockley Hill, Stanmore, Middlesex, UK.

**Introduction:** Proliferative villonodular synovitis (PVNS) is a rare proliferative disorder of the synovium, which most commonly affects the knee, hand, hip and foot joints. The aetiology and cell of origin remains unclear and it is commonly regarded as a benign neoplasm associated with chronic inflammation. Surgery is the mainstay of treatment but this can be difficult in aggressive or recurrent cases. Severe loss of joint function may result or an amputation may be necessary. Preserving function of the ankle joint can be particularly problematic. This report describes a series of cases of PVNS of the ankle and foot treated successfully with conservative surgery and external beam radiotherapy.
**Methods:** Data was collected retrospectively on six patients, treated between 1993 and 2000 at Mount Vernon Hospital. All patients had lesions at the foot or ankle. The median time to treatment following their initial presentation was 19.5 months. External beam radiotherapy was delivered to a dose of 35Gy in 20 fractions over 4 weeks. In all cases parallel-opposed fields were used to encompass the whole joint and affected extra-articular tissue, as defined both clinically and on MRI/CT. Local control and functional ability were assessed for the group.
**Results:** At a median follow-up of 19 months, all six patients remained free from recurrent disease. The treatment was well tolerated with minimal acute toxicity. In two patients, in whom an amputation had been considered it was avoided. In terms of joint function, all but one patient had good range of movement at the ankle and five patients were able to function without the use of walking aids. Follow-up in this series however, was too short to comment on the long-term toxicity.
**Conclusion:** Effective local control can be achieved with conservative surgery followed by external beam radiotherapy in patients with PVNS of the ankle or foot, without significant loss of joint function.

**P80**

**HORMONAL AGENTS ALTER THE RADIO-RESPONSIVENESS OF BREAST CANCER CELLS IN VITRO**

Clare Orridge*[1]; David A.L. Morgan[2]; Stewart G. Martin[1].
[1]Cancer Research UK Academic Unit of Clinical Oncology, University of Nottingham, UK. [2]Department of Clinical Oncology, Nottingham City Hospital NHS Trust, Nottingham, UK.

In the UK, more than 38 000 new cases of breast cancer are diagnosed in women each year. Many patients receive radiotherapy as part of primary loco-regional management. The systemic treatment of breast cancer is often dependant on the oestrogen receptor (ER) status of the tumour, with a large proportion of ER-positive tumours being treated by endocrine therapy, such as Tamoxifen. Previous findings (Paulsen *et al*, 1996) suggest that ER-positive tumour cells treated with Tamoxifen are more resistant to radiation. Tamoxifen is an anti-oestrogenic agent, or SERM (Selective Estrogen Receptor Modulator). This group of compounds bind to the ER and either inhibit the receptor or lead to its partial inactivation. Tamoxifen competes with oestrogen and causes partial inactivation of the ER. Faslodex (ICI-182 780) is a 'pure' anti-oestrogen that has recently been developed. It both down-regulates and inhibits the receptor. Here we report the radio-responsiveness of ER+ve and ER-ve breast cancer cells following exposure to the anti-oestrogenic agents Tamoxifen and Faslodex.
In vitro studies were carried out on MCF-7 (ER+ve) and MDA MB 231 (ER-ve) breast tumour cell lines. Cells were cultured in phenol red deficient, serum-free media to remove any possible influence of oestrogenic substances in the growth media. MTS (proliferation) assays were carried out in the presence of Tamoxifen or Faslodex. Radiation survival (clonogenic) assays were carried out on drug treated and control cells.
Proliferation data indicates that ER-ve cells do not show a significant effect with either Tamoxifen or Faslodex, whereas ER+ve cells, as expected, exhibit decreased proliferation (greatest with Faslodex). Preliminary results from clonogenic assays suggest that as with previous findings, Tamoxifen-treated ER+ve cells appear to be more resistant to radiation. Faslodex however, seems to induce radiosensitisation.
Our data suggests that ER+ve patients receiving radiotherapy should possibly delay starting Tamoxifen treatment. However, patients treated with Faslodex who are exposed to radiotherapy may show an increased response. Experiments are ongoing to examine if normal cell radiosensitivity is also altered.
Reference: Paulsen, G.H.U.; Strickert, T.; Marthinsen, A.B.L.; Lundgren S. 1996. Acta Oncologica, 35 (8), 1011.

**P81**

**INFORMATION GIVEN TO PATIENTS ABOUT ADVERSE EFFECTS OF RADIOTHERAPY: A SURVEY OF PATIENTS' VIEWS**

G.C. Barnett, P.A. Murray, Essex County Hospital, Colchester, UK.

**Objective**: To evaluate the amount of information patients require about adverse effects of treatment before giving informed consent.
**Design**: Structured interview survey of patient opinion.
**Setting**: Clinical Oncology Department, District General Hospital, UK.
**Participants**: 82 adult oncology patients prior to starting treatment with radiotherapy.
**Main outcome measures**: The risks of mild, moderate and severe adverse effects which patients feel are significant and about which they should be informed prior to consenting to treatment.
**Results:** The distribution of responses to the interview was large. For a mild side effect, 31% of patients would only want to be informed if there was at least a 50% chance of it occurring, while 28% of patients wanted to be informed even if the risk of the side effect was as small as 0.1%. For severe side effects, 16% of patients wanted to be informed if the risk was at least 50%, while 44% wanted to be informed of a 0.1% risk. There was no association with sex, treatment intent (radical or palliative), patient age, social class or disease site.
**Conclusions**: It is very difficult to predict how much information patients feel they need prior to giving informed consent. Information needs varied widely within our survey population. Therefore a patient-centred approach must involve tailoring information to individual patient requirements.

© 2002 Cancer Research UK

a blinded fashion by two independent observers. Reduced overall β-catenin expression (<90% of tumour cell positivity) was observed in 73% of sections. Nuclear β-catenin expression was seen in 25% of cases. There was a significant association between positive nuclear β-catenin and both increased MMP-9 expression (≥20% tumour cell positive) and high microvessel counts (upper tertile) (p=0.013 and p=0.030, respectively). Neither nuclear nor reduced overall β-catenin expression was associated with stage, histology or grade of tumour or platelet count. Reduced overall β-catenin expression and positive nuclear β-catenin expression were prognostic on univariate analysis (p=0.045, p=0.0015 respectively). Independent prognostic factors were stage (p<0.001), reduced overall β-catenin (p<0.001), microvessel count (p<0.001) and nuclear β-catenin (p<0.001). These results suggest that nuclear β-catenin acts as a transcription factor up-regulating MMP-9 and as yet undetermined angiogenic growth factors facilitating tumour growth and invasion in NSCLC. Secondarily, disruption of the E-cadherin/ β-catenin complex as evidenced by the prognostic significance of reduced overall β-catenin expression has a role in the pathogenesis of NSCLC.

**P86**

**PLEURODESIS FOR MALIGNANT PLEURAL EFFUSIONS (MPE): A META-ANALYSIS**

*R Agarwal MRCP[1] and Paul Shaw MRCP[2], [1]Institute of Cancer Research, Section of Medicine, Cotswold Road, Sutton, Surrey, SM2 5PT; [2]Sir Michael Sobell House, Churchill Hospital, Oxford.

**Aim:** MPE is a common oncological problem, however despite numerous clinical trials there is a lack of consensus on the optimal method of pleurodesis in these patients. This is due to the small sample size of individual trials published so far and the lack of a comprehensive meta-analysis of the available data. We therefore undertook a meta-analysis of pleurodesis in order to try and define the optimal technique for pleurodesis in patients with MPE.

**Method:** A systematic review of the literature was performed using PubMed, Embase and the Cochrane 'Controlled clinical trials register' from 1980 to 2000. The databases were searched for all articles containing 'pleurodesis' OR 'malignant pleural effusion' as either a textword or subject heading. No other restrictions were imposed on the search strategy. In addition other relevant studies were identified from conference proceedings and by writing to authours of included studies. Only randomized studies of pleurodesis of MPE were included in the final meta-analysis.

**Results:** A total of 33 randomized controlled clinical trials of pleurodesis were identified containing a total of 1318 patients. 4 trials addressed the necessity for a sclerosant for pleurodesis. On meta-analysis the odds ratio for successful pleurodesis was 3.86 (95% CI 1.9-7.9) in favour of the use of a sclerosant. Analysis of the 8 trials comparing the efficacy of talc for pleurodesis to all other sclerosants revealed an odds ratio of 4.73 (2.65-8.42) in favour of talc. Subgroup comparisons of talc with bleomycin and tetracycline were also consistent with this result. No excess mortality was seen on meta-analysis among patients treated with talc. Seven studies compared bleomycin and tetracycline (including doxycycline). On meta-analysis no significant differences in efficacy or mortality among treated patients was observed. Meta-analysis of the comparative efficacy of medical versus surgical pleurodesis is in progress.

**Conclusion:** The results of this meta-analysis show that talc is the most effective sclerosant for pleurodesis in MPE. In addition there is no evidence for excess mortality from the use of talc in these studies. Talc either insufflated or as a slurry should therefore be the sclerosant of choice in these patients.

**P87**

**THE TREATMENT OF LUNG CANCER IN THE ERA OF HAART**

Powles T[1], Hawkin R[2], Shah P[2], Cox S[1], Sarker D[1], Nelson M[3], Gazzard B[3] and Bower M[1].

Depts [1]HIV Medicine, [2]Respirology, [3]Medical Oncology, Chelsea and Westminster Hospital, London.

197 patients with lung cancer have been managed at the Chealsea and Westminster Hospital between 1999 and 2001. These have included six HIV-seropositive patients (5 male:1 female), which had advanced (stage IV) disease. The mean age was 44.2 yrs (range: 313-58). The median CD4 cell count at lung cancer diagnosis was 342 $mm^{-3}$ (range: 117-995), and the median viral load was 283 copies/ml (range: <50-30020). Five patients were on highly active anti-retroviral therapy (HAART) (see table).

| Gender | Histology | Stage | Smoke (PY) | Survival (yrs) |
|---|---|---|---|---|
| Male | Squamous | IV | 60 | 0.7 |
| Male | Adeno | IV | 40 | 0.5+ |
| Female | SCLC | Ext | 30 | 0.1 |
| Male | Squamous | IV | 30 | 0.2 |
| Male | NSCLC* | IV | 40 | 0.2 |
| Male | Broncho-alveolar | IV | Non smoker | 1.2+ |

**unclassified non small cell lung cancer, SCLC = small cell lung cancer, PY = pack years, Ext = extensive.*

All six patients were treated with platinum-based combination chemotherapy using the same protocols that are employed in the immunocompetent patients. The actuarial one-year overall survival is 25%, and there was no significant difference in overall survival compared to the non-HIV patients with lung cancer (log rank p=0.43). Lung cancer in HIV+ patients is not associated with advanced immunosuppression, and thus may be treated in the same fashion as in the general population with similar survival outcomes.

**P88**

**A SINGLE CENTRE EXPERIENCE OF CISPLATIN AND VINORELBINE IN STAGE IIIA-IV NON SMALL CELL LUNG CANCER, (NSCLC)**

J White*, H Glen, A Anthoney, N O'Rourke and D J Dunlop.

Beatson Oncology Centre, North Glasgow University Hospitals NHS Trust, Glasgow, UK.

**Introduction:** Cisplatin combined with vinorelbine has become standard therapy for the management of locally advanced and metastatic NSCLC, (Kelly et al J Clin Oncol 2001. 19;3210-18). As part of budget approval for the use of this regimen, clinicians performed a prospective audit of efficacy and morbidity outcomes.

**Methods:** Data from the first 52 cases with stage IIIa- IV NSCLC diagnosed between 1999-2000, and treated on 2 sites within the Trust are presented here and further data collection is ongoing. Treatment consisted of cisplatin 80mg/$m^2$ d 1 and vinorelbine 30mg/$m^2$ d1 and 8, every 21 days, for a maximum of six cycles. Patients with stage IIIa disease received 2-3 cycles and were then re-assessed for high dose palliative or radical radiotherapy. Some patients received cisplatin and vinorelbine as second line therapy after failure of 1st line non-cisplatin based therapy.

**Results:** The ECOG performance status of patients was 0 = 15.4%, 1 = 53.8%, 2 = 17.3%, 3 = 5.8% and not recorded 7.7%. Stage distribution was 2B = 5.8%, 3A = 5.8%, 3B = 42.3%, 4 = 34.6% and 13.5% not recorded. Significant WHO G3/4 toxicity occurred in 59.6% of cases and haematological g3/4 myelosuppression was complicated by neutropenic sepsis in 21.2% of patients. 17.3% of patients had dose reductions of one or other of the drugs. D 8 vinorelbine was reduced or omitted in 63.5% of patients. Response rates are as follows; CR 1.9%, PR 30.8%, SD 23.1%, PD 21.3%. The OR was thus 32.7%. The median survival was 8.75 months.

**Conclusions:** These response rates and median survival are similar to other published studies. However, in our population of patients, (mostly PS 1-2), significant g3/4 toxicities, particularly complicated neutropenia, but also neuropathy, prompted clinicians to decrease the doses of cisplatin and vinorelbine to 75mg/$m^2$ and 25mg/$m^2$ day 1 and 8, respectively. Further data will be presented.

© 2002 Cancer Research UK

**P89**

**A PROSPECTIVE FEASIBILITY ANALYSIS OF TWO DIFFERENT SCHEDULES OF THE VINORELBINE-CISPLATIN REGIMEN IN ADVANCED NON SMALL CELL LUNG CANCER**.

Gebbia V* (1, 2), Borsellino N (2), Galetta D (3), Caruso M (4), Valenza R (5).
1) Department of Experimental Oncology, University of Palermo ; 2) La Maddalena Clinic for Cancer, Palermo ; 3) Oncological Institute, Bari ; 4) Centro Catanese di Oncologia, Catania ; Oncological Hospital, Palermo, Italy.

The vinorelbine-cisplatin (VNR-CDDP) regimen is considered among the "standard" treatments for advanced poor prognosis stage IIIB or stage IV non small cell lung cancer (NSCLC). However, the VNR-CDDP regimen is not unique since several different dosages and schedules have been developed by different investigators in different countries on the basis of clinical and toxicologic considerations. The classical schedule with cisplatin 100-120 mg/m2 on day 1 plus VNR 25-30 mg/m2/week every 28 days accordingly to the SWOG and the French data is associated with significant hematological toxicity which results in treatment discontinuation in 12-23% of patients and a 30-35% reduction in dose-intensity. These events may negatively conditionate the clinical results of the regimen and give an underestimation of the activity the combinationwhile stressing toxicity.
Based on these toxicologic considerations our group started a prospective phase III study comparing the classical schedule of CDDP 100 mg/m2 on day 1 plus VNR 25 mg/m2/week every 28 days to the schedule of CDDP 80 mg/m2 on day 1 plus VNR 30 mg/m2 on day 1+8 every 21 days. The latter schedule was associated with a >5% discontinuation rate, and with a > 90% dose-intensity. The main aim of the trials was the incidence of severe toxicity, rate of discontinuations, analysis of dose-intensity. Secondary end-points were response rates, time to progression and overall survival. According to a 30% difference of events 100 patients had to be enrolled (50/arm). To date 64 patients eith stage IIIB-IV have been enrolled into the trial and randomized according to stage and performance status (PS 0-1 versus 2). All enrolled patients had to fulfil standard inclusion criteria : age < 70 years, ECOG PS 0-2, measurable disease according to WHO criteria, no major cardiovascular, infective, neurological, renal, or metabolic diseases; absence of CNS metastases. Interim analysis of available data has shown an excess of severe leukopenia (p=0.048), and treatment omissions (p=0.047) in the weekly VNR arm. These data, if confirmed, will cause a re-interpretration of toxicity data achieved in phase III trials reported in medical literature. Final data of toxicity and efficacy parameters are awaited.

**P90**

**AUDIT OF IN-PATIENT MIC CHEMOTHERAPY FOR INOPERABLE NON-SMALL CELL LUNG CANCER**

Giridharan S*, Lo N, Madhava K, Hartley A, Peake D, Rea D
Cancer Centre, Queen Elizabeth Hospital, Birmingham. B15 2TH.

**Introduction:** Chemotherapy has been shown in randomised clinical trials to improve overall survival (OS) in patients with inoperable non-small cell lung cancer (NSCLC). However, patients fulfilling the eligibility criteria for such trials are not representative of the whole NSCLC population and extrapolation of these results to older patients and those with poor WHO performance status (PS) may not be appropriate. The aim of this audit was to assess the OS of a population treated with an in-patient multi-agent regime outside of a clinical trial.
**Methods:** Consecutive patients with inoperable NSCLC receiving in-patient chemotherapy between 1/10/00 and 31/3/01 with the MIC regime (mitomycin 6mg/$m^2$, ifosfamide 3g/ $m^2$ and cisplatin 50mg/ $m^2$ administered every 3 weeks) were included. Age, sex, PS, stage, number of courses, toxicity, radiotherapy details and OS were obtained from hospital, general practice and cancer registry records.
**Results:** 72 patients were identified. At a median follow up of one year 26% of patients were alive. Median OS for all patients was 8.6 months (m). Median OS in patients aged > 70: 11.0m (n=24); aged <70: 7.0 months (n=48); PS 0 patients: 11.0m (n=22); PS 1: 9.4m (n=26); PS 2: 5.1m (n=11); PS 3: 3.5m (n=13). Patients also receiving high dose thoracic radiotherapy (40-50 Gy in 15 fractions): 12.0m (n=13).
**Conclusions:** While the numbers in this study are small, the OS seen in > 70 group suggests that the continued use of multi-agent chemotherapy in selected elderly patients is justified. The OS seen in PS2 patients is consistent with results from randomised trials showing symptomatic but no survival benefit. The poor results obtained in PS3 patients call into question the value of palliative chemotherapy in this group. Further randomised studies with quality of life endpoints are required to define the optimum management for patients in these subgroups.

**P91**

**MALIGNANT PLEURAL MESOTHELIOMA (MPM) AT ABERDEEN ROYAL INFIRMARY, ABERDEEN, SCOTLAND: SURVIVAL AND PROGNOSTIC FACTORS**

Eliyaz Ahamed***,** Marianne Nicolson, Donald Bissett, Keith Kerr and Joe Legge, on behalf on the Aberdeen Lung Cancer Group, Aberdeen Royal Infirmary, Aberdeen AB25 2ZN Scotland

**Object:** In light of the increasing incidence and publication of prognostic factors[1, 2] for MPM, we have audited those prognostic factors and survival in patients diagnosed with MPM between Jan 1994 and Dec 2000 at Aberdeen Royal Infirmary, Scotland.
**Methods**: A retrospective case note review of 78 patients (pts) with histological confirmation of MPM between Jan 1994 and December 2000.
**Results:** Mean age of onset was 65 years (range 41 to 85 years) with a male to female ratio of 4:1. Asbestos exposure was known in 53 and 55 were smokers. Of pathological subtypes 64 were epithelial, 12 sarcomatoid and two were biphasic.71% had local disease while 28% had regional or distant disease. Four pts were treated with surgery, 23 with chemotherapy and 19 patients received palliative radiotherapy. Thirty-two patients received no treatment. The median survival (MS) was 9 months (range 1 to 79 months). Women survived longer than men (11 months compared to 9 months). MS was reduced in pts older than 70 years (6 v/s 11 months) and in those with sarcomatoid mesothelioma (6 v/s 9 months) and it was also less in those with thrombocytosis (7 v/s 8.5), anaemia (5.5a v/s 9 months) and leucocytosis (8 v/s 9). No significant difference was seen in other parameters. LDH was elevated in only two patients.
**Conclusions:** The MS was short but similar to that reported by other groups[3, 4]. Sarcomatoid epithelioma, thrombocytosis and anaemia were important prognostic factors in our survey. Only four pts had surgery and survival was 6,7,9 and 56 months. Chemotherapy did not result in significant survival benefit over best supportive care (MS 9 months). Better therapies are required for this increasingly common and fatal disease.

1 Curran D *et al* J Clin Oncol 1998,16:145-152
2 Herndon JE *et al* Chest 1998 113:723-731
3 Adams VI Cancer 1986 58: 1540-1551
4 Ruffie J Clin Oncol 1989 7:1157-1168

**P92**

**GENETIC INSTABILITY AND NEUROENDOCRINE PHENOTYPE IN BRONCHIAL CARCINOGENESIS: A CLINICAL STUDY**

Marta Ocejo-Garcia[1], Irshad Soomro[2], David R Baldwin[3], Judy M Coulson[1,4], Penella J Woll[1]. [1]CRC Academic Unit of Clinical Oncology and Departments of [2]Pathology and [3]Respiratory Medicine, Nottingham City Hospital, UK; [4]Department of Physiology, Human Anatomy and Cell Biology, University of Liverpool, UK.

Lung cancer is believed to develop through the accumulation of genetic alterations. Deletions at 3p occur earlier than 8p and are frequent in both small cell lung cancer (SCLC) and non-small cell lung cancer (NSCLC). SCLC secretes multiple neuropeptides, which can act as autocrine growth factors for these cancers. Here we investigate whether the neuroendocrine phenotype is an early or late event in bronchial carcinogenesis.
**Materials and methods:** Clinical samples were obtained from lung cancer patients at bronchoscopy (BR, N=10), post-mortem (PM, N=3) and surgery (S, N=12), comprising tumour and bronchial epithelium at 1cm and 4-5cm. Matched normal DNA was obtained from blood (BR) or normal lung (PM, S). DNA and RNA were extracted simultaneously with a Qiagen DNA/RNA kit. Samples were analysed for LOH at 3p and 8p loci by radioactive (alpha $^{32}P$) PCR and gel analysis. RT-PCR was used to detect expression of the neuropeptides gastrin-releasin peptide (GRP) and arginine vasopressin (AVP) using beta-actin as a control. Southern Blotting was carried out to increase sensitivity and specificity. Scanning densitometry was used to quantify peptide expression relative to actin.
**Results:** LOH analysis showed more genetic lesions in the tumour samples compared to the proximal bronchial epithelium (1cm) and none in the distant bronchial epithelium (4-5cm). Surgical samples proved to contain a number of normal cells since GRP was expressed in all surgical samples. GRP expression was increased in tumour samples in 3/3 SCLC patients, whereas 7/8 NSCLC patients showed a decrease or total disappearance of GRP expression in the tumour. AVP was not detected in SCLC post-mortem samples, possibly due to its short half-life. AVP was only expressed in 1/20 NSCLC. Interestingly, a further patient, diagnosed with SCLC 5 years before but with no histological evidence of tumour at the time of bronchoscopy, tested positive for both AVP and GRP.
**Discussion:** These results show that samples of bronchial mucosa adjacent to a lung cancer show increasing genetic instability that recapitulates bronchial carcinogenesis. GRP is expressed in normal bronchial mucosa, but is increased in SCLC and reduced in NSCLC and in some proximal (1cm) samples. AVP was expressed in a small number of SCLC, but not in normal or NSCLC samples. Future work will investigate more patients including a wide range of tumour types to establish whether neuroendocrine changes occur in premalignant bronchial mucosa and may represent a target for chemoprevention.

**P93**

**ENHANCED *IN VITRO* INVASIVENESS AND DRUG RESISTANCE IN A HUMAN LUNG CARCINOMA CELL LINE, DLKP, AFTER TREATMENT WITH MITOXANTRONE, 5-FLUOROURACIL, METHOTREXATE, BCNU, CISPLATIN OR CHLORAMBUCIL, AND ENHANCED DRUG RESISTANCE BUT NOT *IN VITRO* INVASIVENESS WITH VP-16, VINCRISTINE, TAXOTERE OR CCNU**

Yizheng Liang*, Susan McDonnell, Padraig Doolan and Martin Clynes
National Institute for Cellular Biotechnology / National Cell and Tissue Culture Centre, Dublin City University, Glasnevin, Dublin 9, Ireland

Multidrug resistance (MDR) and invasion / metastasis are two major causes of death for cancer patients. Following our previous paper (Liang *et al*., *Eur. J. Cancer*, 37: 1041-1052, 2001) which reported the effects of taxol and melphalan exposure on drug resistance and cell invasion in a human nasal carcinoma cell line, RPMI-2650, we expanded this study to other ten commonly used chemotherapeutic drugs (VP-16, vincristine, taxotere, mitoxantrone, 5-fluorouracil, methotrexate, CCNU, BCNU, cisplatin and chlorambucil) in a human lung squamous carcinoma cell line, DLKP, to investigate the impact of these drugs. The results of toxicity assays showed that all these drugs induced MDR; taxotere and mitoxantrone appeared to be able to induce higher level of MDR. Compared to the DLKP parental cell line, results of RT-PCR showed that mRNA expression of multidrug resistance proteins (MRPs), especially MRP3 and MRP5, was increased in various DLKP drug-resistant variants, along with the overexpression of P-glycoprotein (Pgp) in the DLKP taxotere-resistant variant (DLKP-TXT) as well as breast cancer resistance protein (BCRP) in the DLKP mitoxantrone-resistant variant (DLKP-MITOX). Western blot showed the overexpression of Pgp and MRP1 in DLKP-TXT. Results of invasion assays indicated that exposure to mitoxantrone, 5-fluorouracil, methotrexate, BCNU, cisplatin or chlorambucil made DLKP cells much more invasive *in vitro* while VP-16, vincristine, taxotere and CCNU were not able to induce the invasiveness of the DLKP cells. Gelatin zymography, Western blot and RT-PCR showed that DLKP and its variants secreted matrix metalloproteinase (MMP)-2, -9 and -13. Results of the MMP inhibition assays indicated that MMP-2 played a much more important role than MMP-9 and -13 in DLKP cell invasion *in vitro*, which may have implications for non-small-cell lung cancer (NSCLC) treatment. In conclusion, our results indicate that some effective anti-cancer drugs, especially non-direct-DNA-damaging drugs, do not promote cell invasiveness *in vitro* and could be safer in this regard than other ones, especially some of the alkylating agents, and this may have implications for drug design and drug treatment.

**P94**

**BRITISH THORACIC SOCIETY (BTS) RANDOMIZED FEASIBILITY STUDY OF ACTIVE SYMPTOM CONTROL WITH OR WITHOUT CHEMOTHERAPY IN MALIGNANT PLEURAL MESOTHELIOMA**

W Qian[1], DJ Girling[1] and MF Muers[2] on behalf of all collaborators
[1]MRC Clinical Trials Unit, 222 Euston Road, London NW1 2DA
[2]General Infirmary, Leeds LS1 3EX

*Background*: The incidence of mesothelioma is rising rapidly in Britain. Annual deaths will rise from 1500 in the year 2000 to 3000 in 2020. There is no generally accepted standard treatment. The BTS recommends active symptom control (ASC). It is not known whether chemotherapy in addition prolongs survival or provides worthwhile palliation with acceptable toxicity. Palliation as recorded by patients has been reported for only 2 regimens: mitomycin, vinblastine, and cisplatin (MVP), and vinorelbine (N). The BTS and Medical Research Council plan to conduct a phase III trial comparing ASC only, ASC + MVP, and ASC + N in 840 patients with survival as the primary endpoint. The aim of the present study was to assess (1) the acceptability of the trial design to patients, and (2) the suitability of two standard quality of life (QL) questionnaires for mesothelioma.
*Methods*: Collaborating centres registered all new mesothelioma patients. Those with microscopically and immunohistochemically confirmed disease, any symptomatic pleural effusion under control, a CT scan in the previous month, fit for chemotherapy, and giving signed informed consent, completed EORTC QLQ-C30 + LC13 and FACT-L QL questionnaires, and were randomized between all three or any two of: (1) ASC only, (2) ASC + 4 cycles of MVP, and (3) ASC + 12 weekly doses of N.
*Results*: During one year, 230 patients were registered of whom 109 (47%) were randomized, this being 59% of the 185 eligible patients. 52 from 20 centers were randomized to an option including ASC only. This translates into a rate of 312 patients per year from 60 centers interested in collaborating in the phase III trial. The EORTC QL questionnaire was superior to FACT-L in terms of completeness of data, patient preference, and clarity of definitions of categories.
*Conclusions*: The planned phase III trial is feasible and funding is being sought. The EORTC QL questionnaire has been selected.
Sponsors: The Jane Hancock and Anthony Farmer Mesothelioma Research Funds; Pierre Fabre Oncology; BTS.

**P95**

**A RETROSPECTIVE REVIEW OF TREATMENT OF CERVICAL LYMPH NODE METASTASIS OF SQUAMOUS CELL CARCINOMA FROM AN UNKNOWN PRIMARY**

E Brown*, R Simcock, M O'Connell, F Calman, Guys and St.Thomas' Cancer Centre, London, SE1 7EH, UK

**Introduction** Cervical lymph node metastasis of squamous cell carcinoma from an unknown primary (CUP) form 2-6% of head and neck cancers. Treatment of CUP is controversial and is to be the subject of an EORTC trial (24001-22005). In preparation for this trial we have reviewed our recent patient data.
**Methods** Patients were identified using departmental databases. Patients undergoing treatment with a diagnosis of CUP since 1995 were included. Presentations with Level V nodes excluded. Case-notes were reviewed.
**Results** 31 patients aged 50-79 (median 61.7) were identified with a median follow-up from completed treatment of 21.6 months . All patients had panendoscopy with mucosal biopsies and CT scans of the neck and chest. CT scanning changed the nodal staging in 4 patients. PET scanning was performed in 12 patients and provided additional information in 3 patients. In 2 patients PET suggested possible primary sites (tonsil and nasopharynx) not confirmed on biopsy and in a third patient PET identified a separate NSCLC. 19 patients were initially treated with a neck dissection and 2 patients required neck dissection for residual disease post radiotherapy. Radiotherapy was delivered with radical intent to both sides of the neck and potential mucosal primary sites in 90% of cases. The ipsilateral neck only was treated in 3 cases, in one case with palliative intent. Radical doses prescribed were 60-64Gy in 2Gy daily fractions with electron boosts to residual disease (15 patients) . One patient one died of aspiration during treatment and a second repeatedly failed to attend, all others completed treatment. 6 required

© 2002 Cancer Research UK

admission to hospital for management of acute toxicities. 10 patients have died with a median of 4.5months from completing treatment. 4 patients developed local recurrence, one has been salvaged by surgery the remainder dying (median survival 15 months). One patient developed metastases and died 4 months after treatment. 2 patients developed an identified primary head and neck cancer and one patient died of a metachronous NSCLC. Of the 68% patients alive current disease free survival is 26.5 months**.** All surviving patients have reported xerostomia.

**Conclusions** A policy of pan-mucosal radiotherapy for the majority of our patients with CUP has manageable toxicities and predictable morbidity with over 60% living to 2 years currently. PET scanning did not alter management in this group. The planned EORTC trial is needed to address the controversies over CTV and to provide quality of life data.

### P96
### THE ROLE OF SUPPORTIVE NUTRITION IN RADIOTHERAPY FOR ADVANCED CARCINOMA OF THE HEAD & NECK

*E D Selvadurai and M E A O'Connell
Department of Clinical Oncology, Guy's & St Thomas' Hospital, London SE1 9RT, UK

**Introduction**

The treatment of advanced carcinoma of the head & neck is becoming more complex with combined modality ablative surgery and post-operative radiotherapy (SRT) and combination chemo/radiation therapy (CRT). Treatment breaks due to feeding difficulties adversely affect outcome by prolonging overall treatment time allowing tumour re-population resulting in reduced local control. We studied retrospectively 49 consecutive patients with advanced carcinoma of the head & neck (stages III and IV) treated with radical intent to identify risk factors predictive of those patients requiring enteral nutrition (EN) so that this can be placed prior to RT.

**Patients & Methods**

Data on age, gender, performance status (PS), tumour site, ablative surgery, smoking, radiotherapy field arrangement and size, concomitant chemotherapy and weight loss were collected from the radiotherapy notes.

**Results**

There were 33 males and 16 females, ages 25 to 74 years (mean = 59). Patients were good PS, ECOG=0 (59%) and ECOG=1 (41%). 22 patients required EN: either a nasogastric tube alone (NG=8) or percutaneous endoscopic gastrostomy alone (PEG=8) during or immediately after radical radiotherapy. Another 6 patients received NG first then a PEG. 49% of males required EN compared with 38% females. 54% of patients aged ≥60 required EN contrasted with only 35% of those aged <60. 84% of smokers required EN. 67% of patients requiring EN had tumours located in the oral cavity, oropharynx, nasopharynx and hypopharynx necessitating large opposed lateral radiation portals encompassing >50% of the oral cavity. 35% of surgical patients required EN, 86% of patients receiving concomitant chemotherapy required EN.

**Conclusions**

Factors predicting for significant weight loss requiring EN were: age ≥60, male gender, significant amount of oral cavity and oropharynx in the radiation field, concomitant chemo/radiation therapy and continued smoking during radiotherapy. We are developing an algorithm to determine when EN is likely to be required and ensure that this is in place prior to therapy thus avoiding undesirable treatment gaps.

### P97
### CANCERS OF THE HEAD AND NECK: A NEW HIV-RELATED TUMOUR?

Powles T[1]*, Powles J[2], Nelson M[3], Gazzard B[3], & Bower M[1]
[1]Dept HIV Medicine, [2]Dept ENT Surgery, [3]Dept Medical Oncology. Chelsea and Westminster Hospital, London.

**Background:** Highly active anti-retroviral therapy (HAART) has resulted in an increased life expectancy in people with HIV. Several solid tumours occur more frequently in HIV. The Epstein-Barr virus and the Human Papilloma virus have both been implicated in the pathogenesis of tumours of the head and neck (HNC), and therefore could be expected to occur at an increased frequency in HIV disease. We have examined the Chelsea and Westminster Hospital database between 1987 and 2001, and found 7 patients with head and neck tumours.

**Results:** The median age at presentation was 48 years (range 32-53). All seven were homosexual males and two had an AIDS defining diagnosis prior to their cancer. Six of the patients smoked cigarettes. The incidence of HNC was 1.7 (95% CI: 0.7-3.6) new cases per 10,000 patient years, compared to 0.6 for the HIV-negative males. Treatment with radiotherapy or chemotherapy was poorly tolerated, with marked mucositis (grade 3-4) and a median fall in median CD4 cell count from 131 to 77 $mm^{-3}$. The median progression free survival was 7 months (range: 3-96+) and overall median survival was 27 months. Five of the seven patients died from progressive malignancy and one of HIV.

| Site of cancer | Histopath. | Stage | Nadir CD4* ($mm^{-3}$) | Rx | Survival (months) |
|---|---|---|---|---|---|
| NP | Anapl | T3N0M0 | 280 | RT | 17 |
| NP | Undif | T2N2M0 | 347 | RT | 12 |
| LA | Squam | T1N0M0 | 90 | S | 48 |
| LA | Squam | T3N0M0 | 124 | RT | 27 |
| OC | Squam | T2N0M0 | 250 | S/RT | 96+ |
| OC | Squam | T1N0M0 | 183 | S | 35 |
| OC | Squam | T2N0M0 | 10 | S | 11 |

*Prior to diagnosis of HNC. NP= nasopharyngeal, OC= oral cavity, LA= laryngeal, S= surgery, RT= radiotherapy.

**Conclusions:** In this small series, HNC occurred more commonly in HIV patients compared to HIV negative individuals. The progression free and overall survivals were also markedly worse in the HIV patients. This may be due in part to the poor high toxicity (mucosal and immunological) of the treatment.

### P98
### CHEMORADIATION IN ADVANCED HEAD AND NECK CANCER: HIGH SURVIVAL WITH INDUCTION CHEMOTHERAPY

Martin, WMC*, Premachandra, DJ, Montgomery PQ et al for Norfolk and Waveney Head and Neck Cancer Group c/o Oncology Department, Norfolk and Norwich Hospital, Brunswick Road, Norwich NR1 3SR

PATIENTS AND METHODS: 54 patients with advanced squamous cancer of head and neck (HNSCC) were treated at Norfolk and Norwich Hospital between Dec 95 and Jan 01 by a policy of chemoradiation. Inclusion criteria were patients with stage III – IV or bulky stage 2 tumours, PS 0-1, in whom surgery to the primary would have involved major functional disability. Treatment consisted of induction with 2 or 3 cycles of cisplatin 100 $mg/m^2$ Day 1 with 5-fluorouracil 1000 $mg/m^2$ D2-6 (PF) followed by radiotherapy (RT) to primary and neck. RT dosages were 66Gy/33F to primary tumour with spinal cord shielding after 40-44Gy, or 55Gy/20F for small fields, with 50Gy/25F to uninvolved neck.

Site - distribution was: tonsil 11, posterior tongue 11, other oropharynx 10, pyriform 7, supraglottis 12, glottis 2, floor of mouth 1. Stage – distribution was: II: 4 (7%), 3: 12 (22%), IVA: 24 (44%), IVB 14 (26%). Patients were followed monthly for first year, q 2 months for second year, q 3 months third year, then 6-monthly, with 6-monthly CT from end of treatment. Patients relapsing in the neck (n=3) underwent neck dissection and further adjuvant chemotherapy. Of 4 patients relapsing at the primary site, 3 / 4 underwent radical surgery whereas 1 / 4 showed complete response to further PF chemotherapy. All 7 remain disease free.

RESULTS: Overall survival at 1, 2, 3 and 5 years was 83%, 75%, 79% and 100% respectively and disease - free survival 74%, 72%, 71% and 100% respectively (first 5 patients treated prior to January 1997 have 100% survival).

Of 15 deaths 4 were associated with chemotoxicity. All had N3 disease. 2/4 were after first cycle, 2/4 after 2$^{nd}$ cycle PF (one due to steroid associated perforation). Other deaths were due to recurrence in primary (2), in neck (2), primary and neck (1), metastases (3), unrelated (3). 2 patients have developed second primary tumours. 2 patients are alive with metastases.

© 2002 Cancer Research UK

